# Supplementary material for: Identifying direct risk factors in UK Biobank via simultaneous Bayesian-frequentist model-averaged hypothesis testing using Doublethink
Source: Proc Natl Acad Sci U S A. 2026 Jan 2;123(1):e2514138122. doi: 10.1073/pnas.2514138122 (PMC12773712; doi:10.1073/pnas.2514138122)
Supplement: Supplementary file 1 — Appendix 01 (PDF) [file pnas.2514138122.sapp.pdf]

SI Appendix:
Identifying direct risk factors in UK Biobank via simultaneous Bayesian-frequentist model-averaged hypothesis testing using Doublethink

Nicolas Arning[1], Helen R. Fryer[1], Daniel J. Wilson[1,2,*]

1. Big Data Institute, Oxford Population Health, University of Oxford
2. Oxford University Department for Continuing Education

* Address for correspondence: Li Ka Shing Centre for Health Information and Discovery, Old Road Campus, Oxford, OX3 7LF, United Kingdom. Email daniel.wilson@bdi.ox.ac.uk

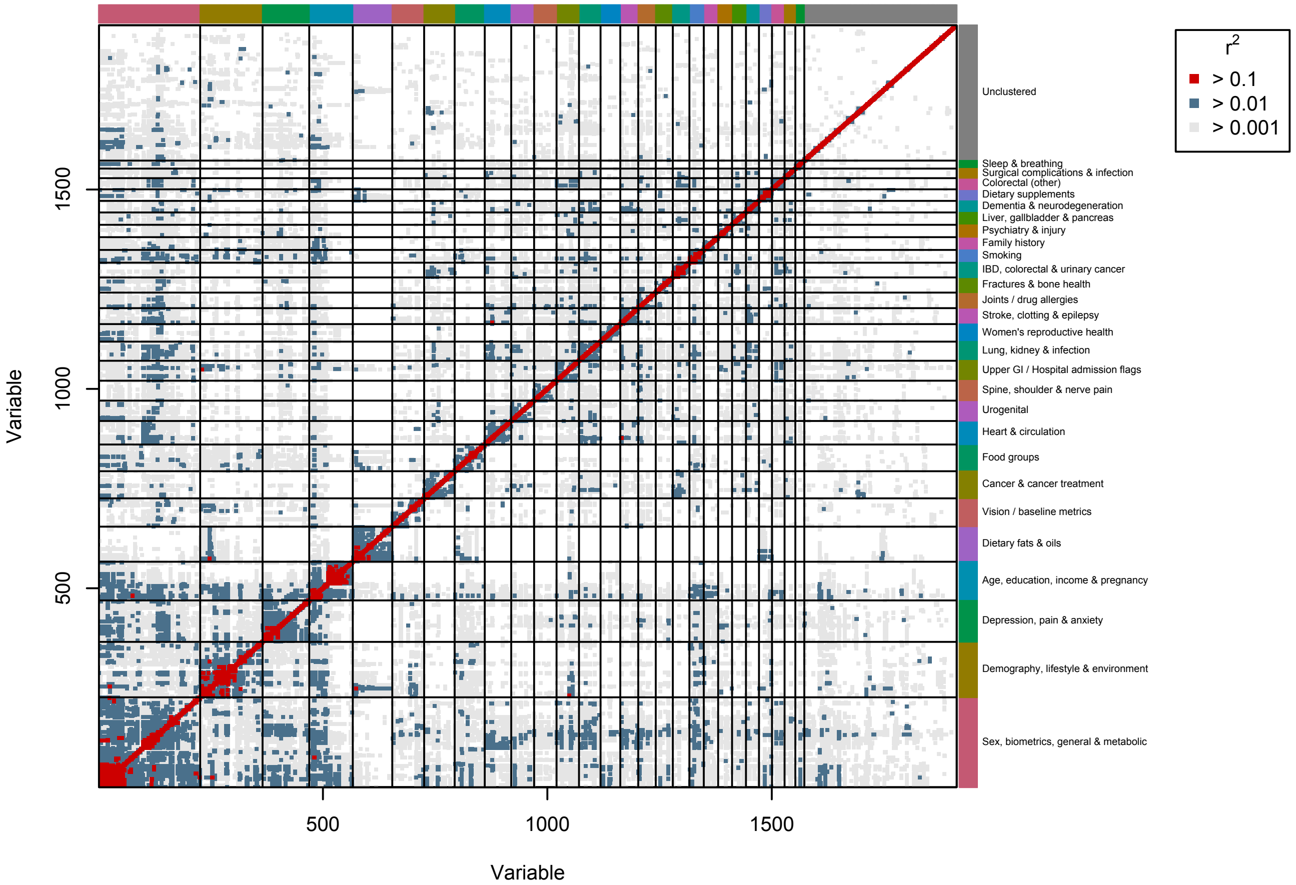

Figure S1 Squared correlation coefficients across the exposome, represented by 1,912 variables in 201,917 UK Biobank participants.

Dataset Descriptions

Dataset S1 Literature review of published papers analysing COVID-19 outcomes in UK Biobank, containing fields from Web of Science and curated with information on the type of analysis, exclusion criteria, and outcomes and exposures included in the abstract and analysis.

Dataset S2 All UK Biobank fields included in the analysis, annotated by field or ICD-10 code, UK Biobank or ICD-10 description and (if a factor) level. Variable clusters are provided alongside.

Dataset S3 Synonyms and categories of variables used in the interpretation of the literature review of published papers analysing COVID-19 outcomes in UK Biobank.

Dataset S4 Squared correlation coefficients r^2 between all pairs of variables. Interactive list of variables in highest r^2 with any named variable.

Dataset S5 Categories applied to results in Table 1, prior groupings, for comparison to the literature review. Variable clusters are provided alongside.

Dataset S6 Categories applied to results in Table 2, post hoc groupings, for comparison to the literature review. Variable clusters are provided alongside.

Dataset S7 Correlates of General comorbidity, defined as variables with squared correlation of 0.001 or greater with any variable assigned to the General comorbidity category in Datasets S5 and S6.

Dataset S8 Results of mediation analysis in which correlates of General comorbidity were removed from the candidate risk factors. Individual variables, pre-defined groups and additional post hoc groups significant with adjusted p-value $p^\star \leq 0.02$. The smallest pre-defined groups significant at $PP \geq 91\%$ are marked in bold. Dashes (-) indicate $p^\star > 0.02$.
